# Operation for Aneurism

**Published:** 1847-02

**Authors:** 


*Operation for Aneurism.*—The following conclusions, as the result of his experience in operations for aneurisms, are drawn by Mr. Guthrie, in a lecture delivered by him at the Royal Westminster Ophthalmic Hospital:

1. That the theory of the operation of aneurism, as dependent on the collateral circulation, cannot be applied with safety to spurious aneurisms of recent occurrence dependent on wounded arteries.

2. That it is inapplicable to wounded and bleeding arteries.

3. That the length of time a spontaneous aneurism has existed is of consequence, as connected with the collateral circulation; although an aneurism should never be allowed to attain that size which may render it injurious to the surrounding parts.

4. The collateral vessels are at all times and under all natural circumstances capable of carrying on the circulation in the upper extremity, whatever disease or injury may affect the principal trunk, provided a due degree of care be taken to maintain the temperature of the part. Whenever the reverse takes place, it is an exception to the general rule.

5. After operations for aneurisms in the lower extremity, the collateral branches are almost always equal to carry on the circulation through the limb.

6. When the principal artery of the lower extremity is suddenly divided, without any previous disease having existed, mortification is not an uncommon occurrence, and is more likely to take place in old than in young persons.

7. When under such circumstances the principal vein is also divided, mortification seldom fails to be the consequence. *Med. Times.—Ibid.*

